# Grading Quality of Evidence and Strength of Recommendations: A Perspective

**DOI:** 10.1371/journal.pmed.1000151

**Published:** 2009-09-15

**Authors:** Mohammed T. Ansari, Alexander Tsertsvadze, David Moher

**Affiliations:** 1 Ottawa Methods Centre, Clinical Epidemiology Program, Ottawa Hospital Research Institute, Ottawa, Canada; 2 Department of Epidemiology & Community Medicine, Faculty of Medicine, University of Ottawa, Ottawa, Canada

**Linked Research Article**

This Perspective discusses the following new Policy Forum published in *PLoS Medicine*:

Kavanagh B (2009) The GRADE System for Rating Clinical Guidelines. PLoS Med 6(9): e1000094. doi:10.1371/journal.pmed.1000094

Brian Kavanagh critiques the GRADE system of grading guidelines, arguing that even though it has evolved through the Evidence-Based Medicine movement, there is no evidence that GRADE itself is reliable.

Evidence-based practice requires translating research findings into clinical and policy decision making. Clinical practice guidelines (CPGs) serve this purpose by evaluating evidence and making recommendations about therapeutic and diagnostic interventions and clinical management strategies. Systematic reviews are considered the best available evidence and are often used in the development of CPGs [1],[2]. Since guideline development involves an assessment of the overall quality of evidence and complex balancing of trade-offs between the important benefits and harms of any given intervention, arbitrariness, value judgements, and subjectivity ultimately come into play in the guideline development process and associated recommendations [3]. In order to minimize cognitive bias in interpreting evidence and make the inherently subjective process more transparent and consistent, CPGs have traditionally employed formal systems or frameworks to understand and grade the quality of the body of evidence and strength of recommendations [4],[5].

One such framework is the grading quality of evidence and strength of recommendations (GRADE), which is commonly used by guideline panels in deriving health care recommendations. GRADE was developed to overcome some of the deficiencies of earlier efforts [6]. GRADE defines the quality of evidence as the collective level of confidence guideline developers have about the validity of estimates of benefits and harms for any given intervention, and the strength of guideline recommendation as the extent of collective confidence that adherence to the recommendation will do more good than harm [7]. It urges guideline developers to consider all important patient outcomes of benefit and harm, to systematically evaluate the quality of their estimates, and to assess the trade-offs between evidence of benefits and harms, the preferences and values placed by patients on outcomes, the opportunity cost associated with the recommendation, and the feasibility of recommendations given a clinical setting before formulating guideline recommendations. Details of the GRADE approach have been published elsewhere [8].

In a new Policy Forum published in this issue of *PLoS Medicine*, Kavanagh [9] questions the external consistency of the GRADE framework by comparing the Surviving Sepsis Campaign (SSC) guideline recommendations developed in 2004 and updated in 2008. Moreover, Kavanagh expresses his concerns on the processes of the GRADE development and its formal validation. Had we likened the GRADE approach to an instrument or a health profile built on discrete logic to capture evidence, we would have concurred with some of Kavanagh's criticism of GRADE. However, we see GRADE as a framework uncovering implicit subjectivity and invoking a systematic, explicit, judicious, and transparent approach to interpreting, as opposed to “capturing” evidence. It reveals how values are assigned to judgments, but what values are assigned it does not dictate simply because it cannot dictate. Below we first present our concern about one aspect of the GRADE framework and then our perspective on the various criticisms of it.

## One Concern about the GRADE Approach

It is arguable that guideline recommendations should be based on cost-effectiveness and opportunity cost analyses. These comparative economic analyses implicitly assume, and therefore do not question, that the allocated health care budget is based on sound evidence to influence guideline recommendations sensibly. In other words, the analyses are supposed to generate reasonable health care policies within a limited budget. However, the appropriateness of the health care budget itself is not established, a priori.

Although the GRADE framework recognizes that guideline panels may legitimately ignore consideration of cost in terms of comparative resource use analysis in developing recommendations, the framework appears to recommend it. This recommendation would be more meaningful if there was accompanying evidence about the appropriateness of allocation of resources towards healthcare in general and a given healthcare discipline in particular.

## Criticisms of GRADE: Our Perspective

- *GRADE is flawed because some guideline recommendations are inconsistent with the evidence, or there is lack of clarity in how they were reached*

Invalid guideline recommendations coming out of CPGs using the GRADE framework do not necessarily imply a problem with GRADE itself. The framework might have been used inappropriately. For example, lack of transparent reporting of how GRADE led to the recommendation, or poor judgment of the quality of evidence, importance of the outcomes for which evidence was found, or benefit-harm trade-offs may be misinterpreted as a flaw with GRADE. Sound use of the GRADE approach by guideline panels is only possible when they are multidisciplinary and include clinical content experts, methodologists, and patients' representatives and have no conflict of interest [10]. Although these are general recommendations, it is unclear whether guideline developers actually ensure a multidisciplinary composition of their panels. For example, eight of the 17 members of the 2007 CPG panel recommending that aprotinin should be used during high-risk cardiac surgery had a financial relationship with the drug manufacturer [11]. Even though evidence of harms of the drug was available from observational studies, the recommendation ignored it. It is to be noted, however, that the panel did not use the GRADE approach.

- *Quality of evidence alone determines the strength of recommendation*

This is incorrect. Recommendations also factor in the potential impact or consequences of the available evidence. Therefore, using GRADE, low quality of evidence can lead to strong recommendation(s) occasionally. For example, evidence from a hypothetical short-term randomized controlled trial with serious risk of bias showing modest benefit of an intervention without serious harms on surrogate outcomes for a fatal infectious disease in a Chinese population might be considered of low quality for whites. However, in a situation of a pandemic, strong recommendation for the intervention in this population is plausible. Another example for a strong recommendation against a treatment may be that of cerivastatin, which was withdrawn from the market due to concerns of drug-related rhabdomyolysis and renal failure based on post-marketing surveillance data [12]. Had a GRADE approach been applied to this low quality of evidence, strong recommendations against the drug would have been made.

- *Changing guideline recommendations or their strength indicates inconsistency, a limitation of the framework*

This is incorrect. Recommendations or their strength, or both, are likely to change if different grading frameworks are used over time. Kavanagh demonstrates this in his comparison of the SSC guideline recommendations developed in 2004 with its 2008 update. Furthermore, recommendations or their strength may change with newer and higher quality of evidence and with repeated more insightful guideline panel deliberations even with the use of one and the same framework, such as GRADE. Also, different guideline panels may come up with somewhat different strengths of recommendations based on the same body of evidence given variation in their clinical and socioeconomic settings and population of interest, and valued judgments. All the above-mentioned should not be grounds for claims that the GRADE is an inherently inconsistent framework that should be discarded.

In our view GRADE should be considered not an instrument for which reliability and validity must be established but rather as a systematic and transparent framework generating recommendations from available evidence as opposed to recommendations originating in an unsystematic and implicit “feel” for it. This attribute of GRADE is a strong argument in favour of using it as it lays the process of translating evidence into recommendations open for scrutiny and criticism by peers and consumers thereby increasing public confidence in guidelines.

- *GRADE-based recommendations, generated for specific population(s) and clinical setting(s), are widely applicable*

We do not believe this to be true. As recommendations are specific to populations and clinical, cultural, and socioeconomic settings, they can be misapplied when these qualifiers are not explicitly and clearly linked to recommendations in summary of guidelines. For example, a recommendation of primary percutaneous coronary intervention in the management of acute myocardial infarction may be less effective than thrombolytic therapy in some developing countries where expertise in and infrastructure for interventional cardiology are lacking. As such, recommendations of guideline panels in developed countries should not be blindly adopted by developing countries without a formal evaluation and vice versa.

- *Once a strong recommendation against an intervention is made as per the GRADE method, future research on it will be stifled*

We believe this is incorrect. Recommendations based on the GRADE approach specifically apply to clinical and not research settings. As long as there is a reasonable rationale, future research on the intervention can continue [13].

Finally, we think that the GRADE framework needs continued discussion and possibly revisions. However, currently the framework is the best available approach to deal with the inherently implicit subjectivity involved in translating.

## Abbreviations

CPGs: clinical practice guidelines
GRADE: grading quality of evidence and strength of recommendations
SSC: Surviving Sepsis Campaign
